# eQTL Epistasis – Challenges and Computational Approaches

**DOI:** 10.3389/fgene.2013.00051

**Published:** 2013-05-31

**Authors:** Yang Huang, Stefan Wuchty, Teresa M. Przytycka

**Affiliations:** ^1^National Center for Biotechnology Information, National Library of Medicine, National Institutes of HealthBethesda, MD, USA

**Keywords:** eQTL, epistasis, genetic association, genetic crosses, network modules

## Abstract

The determination of expression quantitative trait loci (eQTL) epistasis – a form of functional interaction between genetic loci that affect gene expression – is an important step toward the thorough understanding of gene regulation. Since gene expression has emerged as an “intermediate” molecular phenotype eQTL epistasis might help to explain the relationship between genotype and higher level organismal phenotypes such as diseases. A characteristic feature of eQTL analysis is the big number of tests required to identify associations between gene expression and genetic loci variability. This problem is aggravated, when epistatic effects between eQTLs are analyzed. In this review, we discuss recent algorithmic approaches for the detection of eQTL epistasis and highlight lessons that can be learned from current methods.

## Introduction

Epistasis – a form of functional interaction between genes or genetic loci – has emerged as an important factor for the understanding of genotype-phenotype relationships. However, the definition of epistasis varies (Cordell, 2002). Following the work of Bateson (1909), epistasis is often defined as the phenomenon where the effect of a gene on a phenotype is modified by one or several other genes. Currently, epistasis is mostly defined as a non-independent effect of two or more loci on a trait. In the context of quantitative phenotypes, the most commonly assumed model is based on the work of Fisher (1918). Specifically, epistasis is defined as a synergistic effect of alleles of two or more loci when considering their contribution to a quantitative phenotype. Usually the independent effects are assumed to be additive however other models are also used and are not always equivalent (Mani et al., 2008). Alternatives to the Fisher model include non-parametric model-free approaches such as Multifactor Dimensionality Reduction (MDR) or decision tree-based approaches which we briefly describe below. Despite the common assumption that a phenotype is controlled by more than one locus where the effect of loci on the phenotype is non-independent, both definitions are clearly non-equivalent. In particular, Bateson’s definition is based on the requirement that one gene is “acting” while the other gene “modifies,” implying asymmetric roles of two interacting loci. In turn, such asymmetry between genes/loci is absent in Fisher’s definition.

Most phenotypes such as diseases are complex and controlled by multiple loci. Therefore, epistasis needs to be accounted for in genotype-phenotype association studies, potentially allowing an explanation of phenotype variation that single loci associations cannot capture (Zuk et al., 2012). For example, the dependence of the effect of a mutation on the genetic background of an individual is considered a form of epitasis (Lehner, 2011). In addition, epistasis plays a prominent role in many evolutionary processes (de Visser et al., 2011; Lehner, 2011). Furthermore, analysis of epistatic effects might provide functional information about individual genes, uncover functional modules and their mutual interactions (Kelley and Ideker, 2005; Boone et al., 2007; Hannum et al., 2009).

Epistasis might be a consequence of diverse molecular mechanisms including a physical interaction between two genes/proteins where a mutation in one interaction partner may be offset by the other interaction partner. For example, two physically interacting proteins encoded by sex-determining genes *fem-3* and *tra-2* in *C. elegans* species only interact with the partners of the same species due to rapid evolutionary compensation of underlying mutations (Haag et al., 2002). However, such an interaction does not need to be direct. A recent study by Heck et al. (2006) identified such an indirect interaction between two DNA repair genes, MLH1 and PMS1, from a genetic cross of two yeast strains. The resulting progeny that inherited MLH1 and PMS1 from different parents displayed a severe DNA repair defect that was absent in either parent. On a protein complex level, a molecular contact between mutated mitochondrial CYB and nuclear CYTI encoding components of the human cytochrome bcl complex was found to likely restore protein binding (Azevedo et al., 2009). Similarly, functional redundancies may be the cause for epistasis. Gene duplications are considered to be the driving force for such epistatic effects, where intact duplicates participate in back-up circuits. In *S. cerevisiae*, paralogs rescue the organism when their counterparts are mutated. Such functional compensation is achieved through a transcriptional reprograming mechanism where regulatory motifs of duplicates overlap partially with their original counterparts (Kafri et al., 2005; van Wageningen et al., 2010). Furthermore, epistatic effects may represent dynamics of regulatory networks where cooperative interactions and feedback loops contribute to non-linear cellular responses (Lehner, 2011). Although many potential explanations for the emergence of an epistatic effect may exist, the observation of an epistatic effect alone, however, does not suffice to identify a specific underlying molecular mechanism.

Recently gene expression has emerged as a bridge for explaining the relation between genotype and higher level phenotypes such as diseases (Cookson et al., 2009; Schadt, 2009; Kang et al., 2012). Sharing many computational and statistical challenges with GWAS studies, genome-wide expression quantitative trait loci analysis (eQTL) can be empowered by additional genome-wide datasets such as interaction networks (Suthram et al., 2008; Schadt, 2009; Kim et al., 2011). In addition, epistatic eQTL relations might help to pinpoint complex regulatory relationships (Brem et al., 2005; Becker et al., 2012) given that gene expression is often utilized to understand regulatory networks.

**Figure 1:**
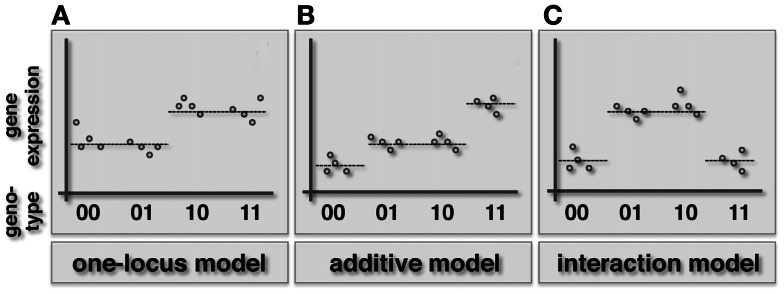
**Schematic illustration of the one-locus, additive and epistatic effects models in haploid organisms**. Specifically, we represent the combined genotype of the pair of loci on the *x*-axis, while the expression level of the underlying gene is shown on the *y*-axis. **(A)** In the one-locus model the genotype of one locus (here the first in the pair) drives the given phenotype. **(B)** In the additive model both loci contribute to the phenotype in an additive way. **(C)** When an epistatic interaction occurs, the effect of the two loci on the trait is non-additive.

Interactions underlying a gene expression phenotype are naturally described by Fisher’s epistasis model (Figure 1). Specifically, two loci *l*′ and *l*′′ in a haploid organism have an epistatic effect on gene *g*, if the expression *y* of a gene is significantly better explained by a widely used synergistic interaction model (Wade et al., 2001; Cordell, 2002).

$$y=b_{0}+b_{1}x^{\prime }+b_{2}x^{\prime \prime }+ix^{\prime }x^{\prime \prime }+\varepsilon$$

than the additive model

$$y=b_{0}+b_{1}x^{\prime }+b_{2}x^{\prime \prime }+\varepsilon .$$

where *x*′ and *x*′′ are the genotypes of loci *l*′ and *l*′′, ε is a noise term and *i* is the interaction term that takes a positive value in the presence of synergistic (or positive) interactions and a negative value in case of antagonistic (negative) interactions.

In a diploid organism the model becomes more complicated by accounting for homo- and heterozygosity and corresponding dominance effects (Cordell, 2002). Consequently in this context, epistasis can be further classified into subtypes such as complementary, dominant, or recessive (Jana, 1971).

The development of methods for uncovering eQTL epistasis has progressed and is accompanied by methods to uncover epistasis in other settings such as case-control studies or organismal level QTL analysis. In particular, Fisher’s model can be adapted to case-control studies by using log odd ratios instead of quantitative traits. In general epistasis detection approaches can be categorized as exhaustive, stochastic, and heuristic (Shang et al., 2011; Van Steen, 2012) (Table 1). As for exhaustive strategies, TEAM computes all two-locus interactions using contingency tables and permutation tests (Zhang et al., 2010b). If two SNPs have the same genotype values for many individuals, computational cost is defrayed by sharing contingency tables. To maximize the sharing of contingency tables TEAM utilizes a minimum spanning tree, where a node is defined as a SNP, and an edge weight denotes the number of samples with different genotypes between different SNPs. To reduce the computational burden, some exhaustive approaches rely on a two-step procedure of screening and testing. For example, BOOST (Wan et al., 2010a) first examines all two-locus interactions in a preliminary screening step where promising SNP pairs are determined through a Kulback–Leibler divergence screen. In a testing stage, likelihood ratio and χ^2^ tests are performed to check if an interactive effect is significant. MDR aims to reduce multi-locus information in a non-parametric and non-model way (Ritchie et al., 2001). In a multistep process, a set of genetic factors is first selected from all pools. Subsequently, the ratio of the number of cases to the number of controls is estimated in each multifactor, two-locus class. In a subsequent step, each multifactor cell is labeled high/low risk if the ratio exceeds/does not exceed a certain threshold. Pooling all high and low risk cells transforms the previous multi-dimensional into a one-dimensional model whose predictive power is finally tested by cross-validation. FastChi (Zhang et al., 2009), Fast ANOVA (Zhang et al., 2008), and COE (Zhang et al., 2010c) use upper bounds to efficiently calculate corresponding test statistics, drastically limiting the search space. Other models include methods that are based on information theoretical considerations (Miller et al., 2009; Hu et al., 2011) and statistical mechanics (McKinney et al., 2007, 2009).

**Table 1:** **Collection of exhaustive, stochastic and heuristic methods for the detection of epistatic interactions in case/control studies or organismal QTL level analysis**.

**EXHAUSTIVE STRATEGIES**	
TEAM	Zhang et al. (2010b)
BOOST	Wan et al. (2010a)
MDR	Ritchie et al. (2001)
FastChi	Zhang et al. (2009)
FastANOVA	Zhang et al. (2008)
COE	Zhang et al. (2010c)
Maximum entropy	Hu et al. (2011), Miller et al. (2009)
Evaporative cooling	McKinney et al. (2009), McKinney et al. (2007)
**STOCHASTIC STRATEGIES**	
BEAM	Zhang and Liu (2007)
epiMODE	Tang et al. (2009)
CART methods	Breiman (2001), Chen et al. (2007), Jiang et al. (2009), Wan et al. (2009), Yoshida and Koike (2011)
SNPInterForest	Yoshida and Koike (2011)
epiForest	Jiang et al. (2009)
MegaSNPHunter	Wan et al. (2009)
SNPHarvester	Yang et al. (2009)
**HEURISTIC STRATEGIES**	
SNPRuler	Wan et al. (2010b)
AntEpiSeeker	Wang et al. (2010)
InterSNP	Herold et al. (2009)
GMM	Isobe et al. (2007)
Adaptive Lasso	Yang et al. (2010)
bNEAT	Han and Chen (2011)
Model based clustering	Wang et al. (2011a)

In a different group of approaches candidates for interactions are determined through stochastic methods. For example, BEAM uses a Bayesian framework to map epistatic effects (Zhang and Liu, 2007), an approach that was generalized to epistatic modules in epiMODE (Tang et al., 2009). In contrast, approaches that are based on classification and regression tree-based methods (CART) (Chen et al., 2007; Jiang et al., 2009; Wan et al., 2009; Yoshida and Koike, 2011) stochastically sample the search space and were used to select pairs of loci for a given trait. In a case-control setting such approaches strive to classify the samples based on genotypic variations. For example, Random Forests (Breiman, 2001) is an ensemble learning method where classification trees are constructed using different bootstrap samples. Specifically, classification trees are constructed by splitting each node, using the best among a subset of randomly chosen SNPs. A classifier is built by aggregating the predictions of all trees where non-sampled data are used for cross-validation sets. Chen et al. (2007) used Random Forests to identify genes that were significant for age-related macular degeneration. In such decision tree forests, SNPs that frequently co-occur in the same tree branch have a propensity for epistasis. This observation was utilized in SNP InterForest, a method applied to the rheumatoid arthritis data from the Wellcome Trust Case Control Consortium (WTCCC) to predict novel interactions (Yoshida and Koike, 2011). Another variant of this approach, epiForest added a sliding window to select a small set of candidate SNPs that were statistically tested for up to three-way interactions (Jiang et al., 2009). In contrast, Mega SNP Hunter uses a tree-based method to divide the whole genome into short subgenomes. While a tree boosting classifier is built on each subgenome, the final classifier consists of a collection of regression trees, where each node represents a SNP, and each path in the trees indicates possible SNP interactions. Ranking all SNPs according to their importance for a disease-control classification process, MegaSNPHunter determines significant interactions along paths in the corresponding trees that involved such high ranked SNPs using *H*-statistics (Wan et al., 2009).

A final group of methods uses heuristics to mitigate the burden of computational cost. For example, SNP Ruler is based on a predictive, non-model-fitting rule inference algorithm to find disease-associated epistatic effects (Wan et al., 2010b). Specifically, a χ^2^ test is used to assess the quality of a rule. To find epistatic interactions, SNP Ruler traverses a set of enumeration tree sets where the nodes of a tree are the genotypes of SNPs, and the path from root to leaf represents allele-specific epistatic effects. To curb the massive burden of traversing a whole tree, SNP Ruler proposes an upper bound of the χ^2^ test to prune the search space. An tEpi Seeker models the search for epistatic interactions as an ant colony optimization procedure (Wang et al., 2010) where each locus is represented by a certain level of pheromones. Assuming that ants communicate through a probability density function of pheromone levels they leave a trace while bouncing from loci, allowing the identification of candidate interactions. Such pairs are tested for the presence of an interaction with a χ^2^ test. InterSNP implements a logistic regression framework as well as log-linear models to allow joint analysis of SNP interactions (Herold et al., 2009). Furthermore, other methods that use heuristics include genotype matrix mapping (GMM) (Isobe et al., 2007) as well as adaptive Lasso (Yang et al., 2010), Bayesian networks (Jiang et al., 2010; Han and Chen, 2011), and model based clustering (Wang et al., 2011a) to select pairs of loci for each expression trait.

Relative to the paucity of methods for the detection of epistasis in case-control studies, genome-wide approaches that have been applied to detect eQTL epistasis are less abundant. Independently of the utilized mathematical or statistical models, a characteristic feature of eQTL analysis is a massive multiple testing problem that emerges from the large number of tests to identify associations between gene expression and genetic loci variability. Furthermore, a large number of eQTLs is often accompanied by weak signals, significantly contributing to both statistical and computational challenges for the detection of epistatic effects. Building in part on the previously discussed methods, two major strategies have emerged. Since testing all possible combinations is statistically and computationally unrealistic, one strategy aims to limit the search space by various filtering strategies. The second group of approaches is specific for eQTL epistasis and leverages modularity of molecular systems. Specifically, statistical power is gained by aiming to identify loci that affect expression of gene groups.

## Selection Based Methods for Detecting eQTL Epistasis

As mentioned above, one of the main obstacles thwarting the effort to detect epistatic effects between QTLs is the large number of loci, which poses both statistical and computational constraints. Specifically, if *g* is the number of genes and *l* is the number of genotyped loci, then the number of possible tests is *g* × *l*^2^ [∼10^15^ for 500 K human SNP array,∼10^19^using dbSNP (built 137), and ∼10^10^ for yeast crosses; Brem et al., 2005]. Such enormous numbers can be reduced by considering genes with expression variability above some user defined threshold, using tag loci to represent a set of markers that are in linkage disequilibrium or markers with a minimum minor allele frequency. In yeast crosses such reduction strategies resulted in ∼1,800 genes and ∼600 loci. Although computationally enumerable, the number of resulting tests (∼10^8^) remains challenging from the perspective of multiple testing. Therefore, exhaustive search by including all possible locus pairs is only feasible in a small number of circumstances (Liu et al., 2011). Therefore, many approaches work with a selected subset of loci instead of testing all possible locus pairs. Boosting both computational efficiency and statistical power we refer to such approaches as selection based methods.

### Biological relevance filtering

One way to reduce the number of tests in eQTL studies is filtering loci based on prior knowledge. Rather than testing all possible pairs (or larger groups) of loci for interactions, only loci that are predetermined to be most relevant/promising based on biological knowledge are selected. For example, Lappalainen et al. (2011) focused on detecting epistasis between variants in coding region and those in *cis*-regulatory regions to study effects of *cis*-regulatory genetic variants on gene expression. Such a local strategy reduces the number of tests dramatically, given that one locus has to be placed in a given gene, while the other locus is located within 1 Mb vicinity. Simply assuming that each dbSNP is assigned to exactly one such neighborhood, such a strategy reduces the number of tests by a factor of ∼10^8^. The significant reduction of the number of tested locus pairs, compared to a brute-force search, allowed them to identify several hundred interactions between *cis*-regulatory elements and corresponding coding regions using eQTL data from the 1000 Genome Project. Analyzing the expression data from transformed lymphoblastoid cell lines of 57 CEU and 56 YRI individuals and restricting attention to SNPs with MAF > 5% and known ancestral state, they identified 433 eQTLs in CEU and 446 in YRI with a false discovery rate (FDR) < 25%. Their results suggested that regulatory and coding variants often modify the functional impact of each other. In addition, eQTLs explaining common disease GWAS signals showed an enrichment of putative epistatic effects, suggesting that some disease associations might arise from interactions, increasing the penetrance of rare coding variants.

In contrast, Becker et al. considered a human *cis-trans* epistatic map. For each transcript the authors considered related *cis* loci and tested them for epistasis with all *trans* loci (Becker et al., 2012). Using the same simplifying assumption as indicated above the reduction factor of the number of tests is estimated to be at least 10^4^. They found that 15% of all transcripts are controlled by significant *cis-trans* locus interactions. Interestingly, no enrichment of genes has been found in close vicinity of trans-SNPs, implying that gene-mediated trans-effects are not a major source of epistasis. Furthermore, some of the genes putatively regulated by *cis-trans* interactions have been previously identified in genome-wide association studies.

Given that one of the simplest explanation of epistasis is a physical or a functional interaction, genes that are known to interact may be more likely to be involved in epistatic effects. Such an assumption suggests a filtering method which focuses on pairs of loci that correspond to interacting gene products (Pattin and Moore, 2008). In case-control studies, Emily et al. (2009) used the experimental knowledge on biological networks to narrow the search for two-locus epistasis. Using such a network based search strategy, four significant cases of epistasis between unlinked loci were identified, that support susceptibility to Crohn’s disease, bipolar disorder, hypertension, and rheumatoid arthritis.

In the context of eQTL epistasis, interaction based filtering methods have been considered by Kapur et al. (2011) who compared their power to the “Both Significant” and “Either Significant” strategies (see below) in yeast and human. In case of interaction based filtering, the authors selected pairs of loci that were close to a pair of genes that encoded two interacting proteins in the STRING database (Szklarczyk et al., 2011). Using the yeast (Brem et al., 2005) and the human dataset (Stranger et al., 2007), they found that the “Both Significant” strategy had the lowest FDR in the yeast data set while the STRING strategy had the worst performance given a predefined *p*-value cut-off. No significant performance difference among the three strategies was found using the human dataset, suggesting that the performance of a method indeed can depend on the data set as suggested by the simulation study of Evans et al. (2006).

Assuming that interacting proteins are a gateway to find epistatic interactions, we estimate that the number of tests are roughly ∼kg^2^ where *k* is the number of markers per gene. In yeast at most one marker per gene exists after accounting for linkage disequilibrium. Most human genes (∼77% or 13,083 genes) have fewer than 10 SNPs (Lehne et al., 2011), suggesting a major numerical advantage by reducing the number of tests by several orders of magnitude in human.

### Data driven filtering

**Figure 2:**
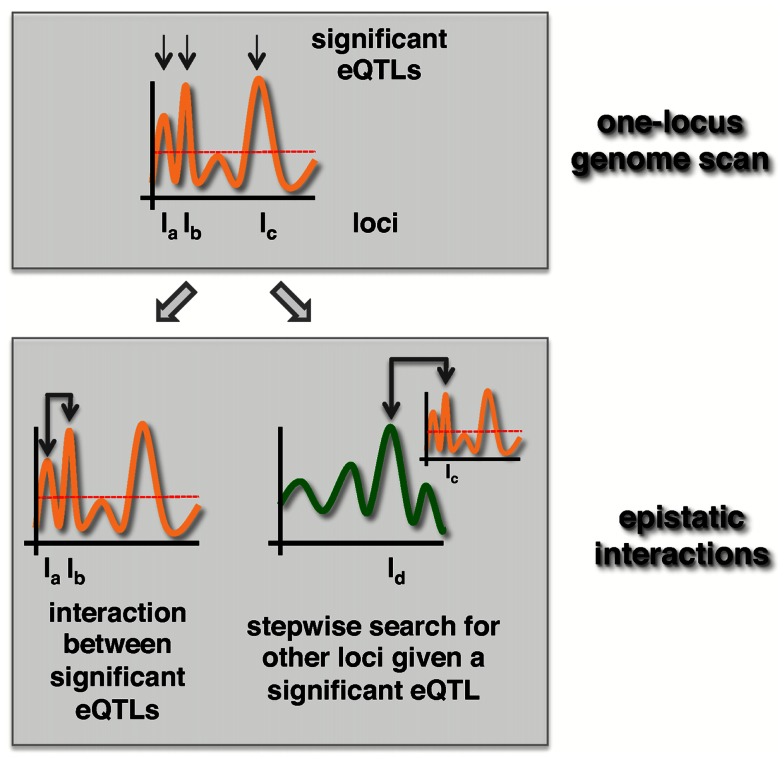
**“Both significant” and “Either significant” strategies to detect epistatic effects**. First, loci with *p*-values that exceed a significance threshold (dotted line) are selected. In the “Both Significant” strategy (left) all pairs of significant eQTLs are tested for the presence of an epistatic interaction in a second step. In turn, loci uncovered in the first step are tested for epistasis with all other loci in the “Either Significant” strategy (right).

The second way of reducing the number of tests is by algorithmic selection strategies. One strategy is to rely on loci with significant marginal effects. The two most popular strategies are the “Both Significant” and “Either Significant” strategy (Evans et al., 2006). Both strategies start by applying one-locus scans to select a relatively small set of loci that are significantly associated with a trait. After selecting a subset of loci that meet a certain significance threshold, the “Both Significant” strategy tests for interactions between all possible pairs of loci in the selected subset. In turn, the “Either Significant” strategy performs another one-locus genome scan conditional on the initially found loci (Figure 2). In other words, this strategy considers possible interactions between a locus in the subset and all other, providing at least one locus with a marginal effect. Similar to this two-step procedure that allows the identification of epistatic interaction between a locus pair (one locus in one step), higher-order interaction can be detected with three-step or four-step genome scans (Stich et al., 2007; Pettersson et al., 2011).

As for examples, Marchini’s et al. (2005) work employed the “Both Significant” strategy while Yang’s et al. (2007) approach used the “Either Significant” strategy with different variations. In the first step, a technique called marker pair selection was used (Piepho and Gauch, 2001) to select a set of genomic intervals that were each labeled by a pair of markers. Such intervals that were defined by a subset of markers narrow the search space for QTL detection. In the next step, the presence of an interaction effect between two intervals was tested, and two interacting intervals were added to a candidate interval set. Finally, a search for epistasis between two loci located on intervals in the candidate set was performed using regression models where all intervals in the candidate set were used to model QTL effects outside the two loci being tested.

However, the question remained which strategy works better for a given scenario. Evans et al. (2006) approached the problem by applying both strategies to detect epistasis on simulated data. As expected, the ability to detect interactions by the “Both Significant” strategy tends to decrease as the threshold for selecting the first locus becomes more stringent. In contrast, the performance of the “Either Significant” strategy was more dependent on the data simulation model, suggesting that its power might also depend on the underlying real data. Similarly, Wei et al. used simulated (diploid) data to compare a 1D scan – a variant of “Either Significant” strategy – and the full 2D scan. Evaluating their simulation results they found that the relative power of each method depended on the type of epistasis (Wei et al., 2010).

As mentioned above, both strategies can be readily applied to eQTL-based epistasis. However, the number of gene expression traits in eQTL studies is significantly higher relative to the number of phenotypes in classical QTL studies. Such numbers contribute to a more severe multiple testing issue and prompt specialized approaches targeting eQTL data. Although new approaches use the “Both Significant” or “Either Significant” strategy, various techniques are utilized to estimate FDR more accurately. Storey et al. applied a variant of the “Either Significant” strategy to detect epistasis in yeast eQTL data for 112 yeast F1 segregants (Brem et al., 2005). Instead of selecting a set of loci for an expression trait first, only the most significant locus for the trait was considered. Subsequently, a secondary locus was selected that provided the largest improvement in statistical power, comparing a two-locus interaction model to a one-locus model given the primary locus. Considering 613 equally spaced loci and focusing on parts of loci from different chromosomes they found that there were 3,540 traits significantly linked to a locus pair at a 5% FDR threshold. Furthermore, they were able to show that epistatic effects contributed to gene expression variation in at least 14% of all expression traits (Storey et al., 2005).

The reduction in the number of considered loci-gene triplets strongly depends on the significance cut-off used for the marginal effect. Assuming that on average one association is selected per gene in the first step, the Either Significant strategy would reduce the number of considered triplets from *g* × *l*^2^ to *g* × *l*.

A variant of such a two-step approach was generalized to more than two loci and applied to eQTL data by Zou and Zeng (2009). Specifically, they applied sequential genome scans to detect a set of loci for each expression trait. Each scan searched for one locus, which was conditional on loci already identified in previous scans without considering interactions. Focusing on a trait, they tested for interaction effects among all identified loci. Applied to yeast eQTL data (Brem et al., 2005) more eQTLs were found compared to Storey et al.’s results. However, fewer interactions were finally detected with a similar FDR threshold.

**Figure 3:**
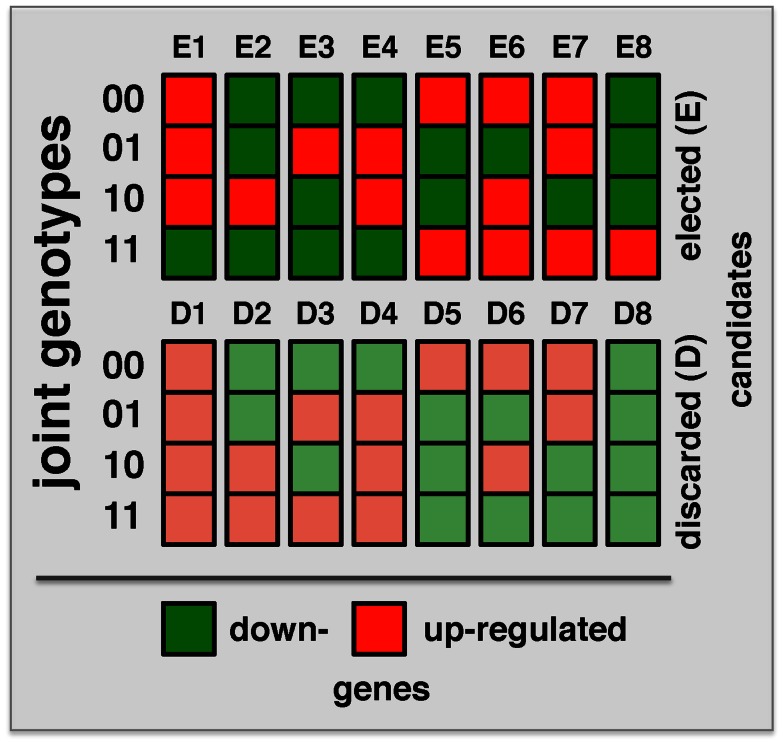
**Combinations of binary locus genotypes and binary expression levels make up 16 different configurations**. Binary numbers on the left refer to the locus genotype while the colors of the squares indicate gene expression levels (low/high). The method uses expression pattern for loci 00/01/10 to predict expected expression level for pair 11 under single locus or non-interaction model. For example, configurations E4 and E5 correspond to “XOR” function where the expression patterns depend on whether both loci are inherited from the same or from two different parents.

“Both Significant” and “Either Significant” strategy facilitate the detection of interactions with one or both loci that have a relatively significant marginal effect. To detect interactions where both loci lack such an effect, a strategy is needed that allows the simultaneous step-wise selection of two loci. A recently developed strategy, Symmetric Epistasis Estimation (SEE) is a two-step method applicable to haploid cases. SEE utilizes general patterns of expression and genotype that are expected to be enriched with epistatic effects. Specifically, 16 possible combinations of the genotypes of a pair of loci and expression state of a gene after discretizing gene expression data into up and down states were defined (Figure 3). The method tests if the expression for locus genotype combination 11 can be inferred from the expression patterns in locus genotype 00/01/10, using either single locus or independent effect regression models. In this way, among these 16 patterns, eight were discarded as consistent with the expectation. Repeating this argument after switching the roles of 0/1 allows for additional filtering out of configurations E1 and E8 since expression pattern for 00 could be predicted from 01/10/11 using independent model despite the fact 00/01/10 are not informative for the prediction of 11. Triplets passing the filtering criterion for large enough set of progenies were determined using a graph theoretical approach (Huang et al., 2012). Obviously, passing the filtering criterion does not imply interaction and therefore selected triplets were further tested for epistasis using Fisher’s model. Similarly, filtered triplets might contain false negatives as binarization of gene expression hides more subtle expression patterns. SEE was applied (without the 0/1 swap) to 34 progeny crosses of *P. falciparum* (Gonzales et al., 2008), identifying 3,796 epistatic triples (two loci and expression trait) with FDR < 0.003. Compared to the “Either Significant” strategy, this method allowed the detection of more interactions with a smaller FDR. As expected, the overlap of interacting pairs that were obtained with both methods was very small. Since the same Fisher model was applied in each case the observed difference was mostly a result of the difference in the filtering approach. Indeed, triplets selected by the SEE approach typically didn’t have significant marginal effects. Specifically, strong marginal effect and strong symmetric pattern are hard to detect when the number of progenies is small. In conclusion, SEE and the “Either Significant” strategy explore different subspaces of the epistasis landscape, indicating the importance to consider both approaches.

## Gene Module Based Methods

Molecular systems are increasingly recognized as being modular (Eisen et al., 1998; Stuart et al., 2003; Barabasi and Oltvai, 2004; Wagner et al., 2007). Such patterns imply that a locus or a pair of interacting loci is likely to impose effects on a group of genes than just a gene alone. Such a perspective is consistent with the existence of eQTL “hot spots,” defined as loci that affect a larger group of expression traits (Breitling et al., 2008). Therefore, several methods focused on the epistatic effect on a group of genes that respond in a consistent way to a genotypic variation rather than identifying epistatic effect of a pair of loci on a gene’s expression. Such a module based approach increases statistical power but requires a definition of a module as well as its phenotype. In a naïve approach, genes with similar expression profiles may be clustered where aggregated expression profile of genes in such clusters may represent the underlying phenotype. However, such an approach does not provide other obvious advantages than reducing the number of tests for epistasis.

**Figure 4:**
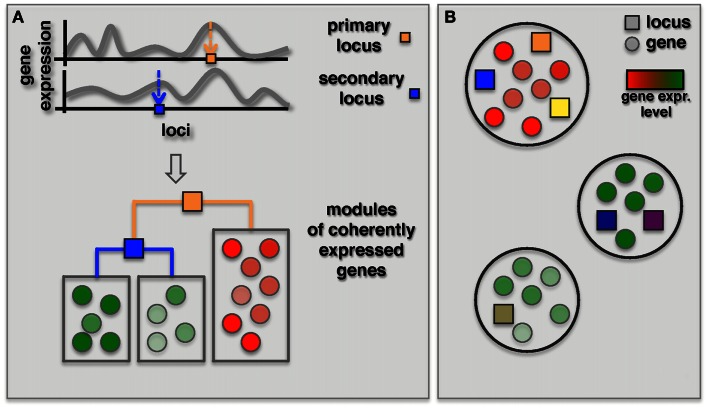
**Identification of co-expressed modules jointly regulated by two or more loci**. **(A)** The GOLPH algorithm starts with building a decision tree to classify genes associated with a primary locus (orange square) according to their expression. The primary focus is placed in the root of this tree while branches correspond to the alleles. Other decision nodes are selected from the remaining set of loci. Subsequently, additional genes consistent with the decision tree model are included. **(B)** Bayesian partition method simultaneously identifies co-expressed modules and their regulating loci using a MCMC approach. Genes in each module are represented by circles colored according to their gene expression and loci are represented by squares where different colors represent different loci.

As an alternative, two recent methods focused on the simultaneous identification of interacting loci and regulated modules. The GenOmic Linkage to PHenotype (GOLPH), method proposed by Litvin et al. (2009), can be considered a clever “modularization” technique that combines a two-step approach as described in the previous section with a decision tree strategy (Figure 4A). In the first step, GOLPH identifies hotspots i.e., loci that are associated with a group of genes. The hotspot locus is considered to be the primary locus and subdivides the population of genes into two allele-specific groups. For each of these groups GOLPH tests the existence of a second regulatory locus. Finally, the resulting decision tree is used to expand the initial “hotspot” module to include other genes whose expression is consistent with the current module specific genes. A pair of loci identified as regulators of a module can then be tested for epistatic effects. While the method pinpointed some epistatic modules, the majority of modules notably showed allele-specific interactions where the secondary locus was modifying the effect of the primary locus only for one of the two alleles.

Complementing the primary-secondary locus approach, Bayesian models were introduced for the detection of epistatic modules (Zhang and Liu, 2007; Tang et al., 2009; Zhang et al., 2010a). Specifically, Zhang et al. (2010a) proposed a Bayesian partition method where a module and its regulating loci are identified simultaneously without requiring the identification of a primary locus (Figure 4B). In this method a component of a partition is defined as a set of genes. Associated loci and components were then identified using an MCMC approach. Finally, they used the first principle component of gene expression traits of the module as dependent variable to test for epistasis between loci for each identified component with more than one locus. Interestingly, the authors found that three out of nine modules with two loci actually had an epistatic effect on the genes in the module. In comparison, we found, similarly to the reduction based methods, that none of the above three modules overlapped with 18 modules with epistatic effects that were identified by Litvin et al. (2009). However, one of the three modules had a significant overlap with an interaction hotspot discovered by Brem et al. (2005).

## Outlook and Conclusion

In recent years, several strategies have been developed to detect pairs of loci that have epistatic effects on gene expression traits. Overcoming loss of statistical power and computational cost of exhaustive testing, these methods leverage data characteristics and algorithmic strategies to identify interacting pairs with confidence. However, each of these different strategies appear to bias the results toward the detection of certain classes of interactions at the expense of other classes. Therefore, delineating a comprehensive landscape of epistasis will require the combination of several prediction strategies.

The paucity of uncovered epistasis depends not only on the method and the definition of epistasis, but also on the dataset. For example, in our study (Huang et al., 2012) we found that the same method, allowing us to identify abundant presence of epistatic interactions in the population of progenies in plasmodium reported much smaller number of interactions in yeast. Such a difference might relate to the fact that the parental strains of *P. falciparum* are under strong evolutionary pressure to adapt independently to the host environment. Indeed, the most frequent epistatic effects we identified were consistent with configurations E4 and E5 in Figure 3. Note, that in these configurations gene expression is similar if the two loci are inherited from the same parent but changes when loci were inherited from both parents. Such an inheritance pattern can disrupt the interaction properties that evolved independently in each parent. In contrast the standard laboratory strain and a wild isolate from a California vineyard that were used for obtaining progenies in yeast crosses (Brem et al., 2002) represent progenies of two strains that adapted to somewhat different environmental circumstances, potentially impacting different distribution of interaction types observed in these crosses.

Finally, we note that the debate about the biological importance of statistical epistasis has been controversial. Skepticism is fueled by the difficulty of linking such effects to biological causes and lack of reproducibility in the context of disease-control studies. As indicated in the introduction, the presence of epistasis does not imply a specific underlying molecular mechanism, prompting the question whether it is reasonable to strive to identify one. In the context of epidemiological studies of disease risk it has been argued that making inferences about biologic interactions from statistical interactions is not straightforward and even sometimes inappropriate (e.g., Thompson, 1991; Greenland, 2009). In addition, Wang et al. (2011b) questioned the importance of the interaction term and argued that statistically modeled interaction and main effect terms should not be separately interpreted to discover biological interactions.

Do those concerns diminish in the context of eQTLs when the trait in question is gene expression? While, admittedly, these concerns in eQTL analysis remain open, new avenues of investigation hopefully lead to a better understanding of related issues. The fact that eQTL analysis involves simultaneous examination of thousands of traits is not only a statistical obstacle but also provides important benefits. First, it allows for meta-analysis, uncovering properties of the previously mentioned *cis-*gene and *cis-trans* pairs (Lappalainen et al., 2011; Becker et al., 2012). Since gene expression is a molecular trait, eQTLs can further be naturally combined with other molecular data such as physical or functional interactions. Such auxiliary data supports increased interpretability of the results, therefore providing a platform for the generation of testable hypotheses. Finally, modularity of biological systems can be leveraged to reduce false positives and to increase interpretability of the gene expression data by leveraging functional associations of genes in a module. While these considerations do not resolve the initial concerns, new high-throughput functional measurements and the emergence of systems approaches open unique opportunities to gain a better understanding of these issues (Phillips, 2008). Given the limitations of individual methods, the delineation of a comprehensive landscape of putative interactions will require a set of complementary methods. We believe that further development of biologically motivated approaches to identify eQTL epistasis, such as methods that leverage modularity of biological systems and connect eQTL analysis to particular organismal phenotype, will continue to provide insights into the regulation on single gene and gene modules.

## Conflict of Interest Statement

The authors declare that the research was conducted in the absence of any commercial or financial relationships that could be construed as a potential conflict of interest.
